# Different patterns of weight bearing impact sagittal spinal balance

**DOI:** 10.1186/1748-7161-7-S1-P2

**Published:** 2012-01-27

**Authors:** HR Weiss

**Affiliations:** 1 Orthopedic Rehabilitation Services, Gensingen, Germany

## Background

A physiologic sagittal alignement of the spine with lumbar lordosis and thoracic kyphosis is the most stable position of the spine , while in patients with Idiopathic Scoliosis (IS) the sagittal profile of the spine is flattened or inversed [1,2]. It has been shown that a correction of the sagittal profile also corrects coronal plane deformity in patients with IS [1]. Therefore sagittal corrections seems to play an important role in the conservative treatment of IS. Within the ‚Best Practice’ PT program simple tools are used to correct scoliosis in 3D. One of these tools is the sagittal realignment of the scoliotic pattern of weight bearing. The impact different weight bearing (WB) patterns might have are subject of this investigation.

## Materials and methods

13 healthy subjects (females only, age range from 18 to 45 years) have been investigated with the help of suface topography (Diers® Formetric) in two different patterns of weight bearing (WB forefoot / WB heel). Kyphosis angle, lordosis angle and the inflection point (IP) between the lordotic and the kyphotic curve have been investigated.

## Results

There was a significant increase of lordosis angle (49,5° to 51,1°; p = 0,047) in WB forefoot. No change of kyphosis angle has been detected. IP had a tendency to slip more cranially, however this was not significant.

## Conclusions

WB on the forefoot increases lordosis angle and by this stabilizes the spine. The different patterns of WB do not seem to change the angle of thoracic kyphosis.
